# Dietary Supplementation with Green Alga (*Chlorella pyrenoidosa*) Enhances the Shelf Life of Refrigerated Nile Tilapia (*Oreochromis niloticus*) Fillets

**DOI:** 10.3390/foods14091642

**Published:** 2025-05-07

**Authors:** Leticia Franchin Rodrigues, Mayumi Fernanda Aracati, Susana Luporini de Oliveira, Camila Carlino-Costa, Romário Alves Rodrigues, Mateus Roberto Pereira, Hirasilva Borba, Cleber Fernando Menegasso Mansano, Gabriel Augusto Marques Rossi, Jorge Galindo-Villegas, Gabriel Conde, Luiz Arthur Malta Pereira, Hélio José Montassier, Marco Antonio de Andrade Belo

**Affiliations:** 1 Department of One Health, Sao Paulo State University (UNESP), Jaboticabal 14884-900, SP, Brazil; leticia.franchin@unesp.br (L.F.R.); mayumi.aracati@unesp.br (M.F.A.); susana.luporini@unesp.br (S.L.d.O.); camila.carlino@unesp.br (C.C.-C.); romario.a.rodrigues@unesp.br (R.A.R.); mateus.roberto@unesp.br (M.R.P.); hirasilva.borba@unesp.br (H.B.); helio.montassier@unesp.br (H.J.M.); 2 Animal Nutrition Department, Brazil University, Fernadópolis 15600-000, SP, Brazil; cleber.mansano@ub.edu.br; 3 Microbiology Department, University Vila Velha (UVV), Vila Velha 29102-770, ES, Brazil; gabriel.rossi@uvv.br; 4 Department of Genomics, Faculty of Biosciences and Aquaculture, 8020 Bodø, Norway; jorge.galindo-villegas@nord.no; 5 College of Veterinary Medicine, University of Araraquara (UNIARA), Araraquara 14801-340, SP, Brazil; ga_conde@hotmail.com; 6 Animal Production Program, Brazil University (UB), Descalvado 13690-000, SP, Brazil; luiz.pereira@ub.edu.br; 7 Laboratory of Animal Pharmacology and Toxicology, Brazil University, 950 Hilário da Silva Passos Avenue, Jardim Universitário, Descalvado 13690-000, SP, Brazil

**Keywords:** chlorophyta, microalga, antioxidant, food quality, fish flesh

## Abstract

Given the critical need for strategies to enhance food safety and quality, particularly due to the rapid spoilage of fish, this study investigated the effect of dietary supplementation with green alga (*Chlorella pyrenoidosa*) on the shelf life of Nile tilapia (*Oreochromis niloticus*) fillets. In addition, the microbiological, physicochemical, and sensory qualities of the fillets were evaluated. Eighty-four healthy tilapias (100 ± 2 g) were randomly allocated in three groups (*n* = 28 per group): a control group (without *C. pyrenoidosa* supplementation) and two treatment groups supplemented with either 5 or 10 g of *C. pyrenoidosa* per kg of feed. After 30 days of feeding, the fish were slaughtered and their fillets stored at 4 °C for evaluation at 0, 7, 15, and 30 days post-slaughter (dps). Fillets from fish supplemented with *C. pyrenoidosa* showed significant lower counts of coliforms, Enterobacteriaceae, and mesophilic and psychrotrophic microorganisms compared to the control. Supplementation also reduced thiobarbituric acid reactive substance (TBARS) levels and pH over the 30-day storage period. Sensory and color analyses indicated improved brightness, appearance, and firmness in fillets from treated groups, while control fillets exhibited poorer odor quality. Our findings demonstrate a dose-dependent effect of *C. pyrenoidosa* supplementation in improving the microbiological, physicochemical, and sensory quality of Nile tilapia fillets during refrigerated storage for up to 30 days.

## 1. Introduction

The shelf life of food products is influenced by multiple factors, including storage duration, environmental conditions such as temperature, and the intrinsic susceptibility of the product to quality degradation. Food spoilage occurs because of physical, chemical, and biological changes that can compromise the nutritional value, safety, and sensory attributes of the product [1]. In fish, spoilage is characterized by the development of off-odors and flavors, mucus accumulation, gas production, abnormal coloration, and texture deterioration. These changes emerge as a product of autolysis, oxidation, bacterial activity, or a combination of these processes, directly impacting the shelf life of fish [2].

To extend shelf life, strategies that prevent or reduce oxidative damage are essential, with antioxidants playing a key role in mitigating both oxidative and microbial spoilage in freshwater seafood products [3,4]. *Chlorella pyrenoidosa* is a green microalga widely used as a nutritional supplement for both humans and animals [5]. Its characteristic green color is attributed to the high concentrations of chlorophyll-a and chlorophyll-b, as well as other pigments such as carotenoids (α-, β-, and γ-carotene) and xanthophylls [6]. Dietary supplementation with carotenoids is an increasingly common practice in aquaculture, as it enhances carcass quality and antioxidant capacity in fish [7]. Additionally, carotenoids have been shown to improve the microbiological, physicochemical, and sensory properties of seafood products [8].

In aquaculture, dietary inclusion of *Chlorella* has been shown to enhance fish health and improve the quality of flesh by modulating antioxidant capacity, immune function, and pigmentation. Several studies have reported that microalgae supplementation can reduce lipid oxidation, inhibit microbial spoilage, and improve the texture, color, and sensory characteristics of fish fillets during storage. For example, Takyar et al. [4] demonstrated that *Chlorella vulgaris* and *Spirulina platensis* extracts extended the shelf life of rainbow trout by improving oxidative stability. Similarly, Rosas et al. [7] showed improved antioxidant performance and fillet coloration in mullets fed with carotenoid-rich microalgae. These findings highlight the potential of *Chlorella pyrenoidosa* as a natural feed additive for improving the microbiological, physicochemical, and sensory quality of fish flesh, making it a promising candidate for sustainable strategies in aquaculture and food preservation. Given the nutritional importance of fish in ensuring food security and the need for effective preservation strategies, this study aimed to evaluate the effect of dietary *C. pyrenoidosa* supplementation, administered orally through feed, on the microbiological, physicochemical, and sensory quality of Nile tilapia (*Oreochromis niloticus*) fillets during 30 days of refrigerated storage.

## 2. Material and Methods

### 2.1. Animals

A set of 84 healthy Nile tilapia (*O. niloticus*), each weighing approximately 100 g, were purchased from the same spawning and placed in 12 aquariums (*n* = 7), with each aquarium having a capacity of 100 L of chlorine-free running water from an artesian well, flowing at a rate of 1 L/min. Prior to the start of the experiment, the fish were monitored for physiological condition and general behavior to ensure the inclusion of only clinically healthy individuals. Animals were considered suitable if they exhibited species-specific body coloration, absence of external lesions, intact scales, clear eyes, balanced swimming, responsiveness to stimuli, and no signs of stress such as lethargy, erratic swimming, or prolonged bottom-dwelling behavior. This screening ensured the homogeneity of the experimental groups and the reliability of subsequent results. The fish were acclimated for 15 days to allow their plasma cortisol concentrations and osmolarity to return to baseline levels. During the initial three days of acclimation, the fish were treated with a NaCl solution at a concentration of 6.0 g/L [9]. They received commercial pelleted feed (32% crude protein), constituting the basal diet. The animals were fed three times a day, corresponding to 2% of the biomass of the aquariums. Water quality was monitored at feeding times throughout the experimental period using a YSI-63 pH meter (YSI incorporated^®^, Yellow Springs, OH, USA) and a YSI-55 oximeter (YSI incorporated^®^, Yellow Springs, OH, USA) and its values remained within the appropriate range for the welfare of tropical fish [10] (dissolved oxygen = 4.07 ± 0.89 mg/L; temperature = 27.64 ± 2.05 °C; pH = 7.64 ± 0.54; and electrical conductivity = 208.29 ± 97.57 μS/cm).

### 2.2. Experimental Design

Tilapia were randomly distributed among 12 aquariums (100 L of water each, *n* = 7) with the following treatments: T0 (no treatment with *C. pyrenoidosa*), T1, and T2 (treated with 5 g and 10 g of *C. pyrenoidosa* per kg of feed, respectively). After one month, the fish were slaughtered, and fillet samples were collected for analysis of microbiological, physicochemical, and sensory characteristics. These analyses corresponded to the following post-slaughter periods: 0, 7, 15, and 30 days post-slaughter (dps) (*n* = 7 per period, totaling 28 tilapia per treatment).

### 2.3. Experimental Diets

The basal diet composition consisted of commercial pelleted feed (LAGUNA^®^, ADM Company, Sao Paulo, Brazil), containing 32% crude protein, 7% ether extract, 5% crude fiber, and 12% mineral matter. The animals were fed three times a day, at 8:00 am, 1:00 pm, and 6:00 pm, corresponding to 2% of the aquarium biomass. To standardize the experimental diet containing *C. pyrenoidosa* (Calêndula Vet Pharmacy, Sao Carlos, SP, Brazil), 5 and 10 g/kg of feed were added to the commercial feed, following the study by Abdulrahman et al. [11]. For diet preparation, the commercial feed was weighed based on the average biomass per tank, and 2% soybean oil was incorporated along with the respective amounts of *C. pyrenoidosa* (5 or 10 g/kg). The control diet received 2% soybean oil without microalgae to ensure energetic balance and consistency in pellet coating across treatments. The diets were stored in dark plastic bags and kept at 9 °C for use during the experimental period of one month prior to the slaughter of the animals. After the 30-day feeding period, no significant performance variation was detected across dietary groups: control fish gained 26.1 g, whereas those supplemented with 5 g and 10 g kg^−1^ of *C. pyrenoidosa* presented 34.1 and 31.7 g, respectively.

### 2.4. Slaughter of Fish and Removal of Fillets

The stunning method employed was hypothermia, using a mixture of hyperchlorinated water (5 ppm) and ice in a 2:1 ratio. This mixture was placed in a 15 L Styrofoam box lined with sterilized plastic bags, into which the fish were immersed for 3 min to induce stunning. The plastic bags and knives were changed for each group to prevent cross-contamination. After the fish were rendered unconscious, decerebration of the brainstem was performed. The filleting process was carried out by a single individual, who divided each fish into two fillets (right and left, weighing about 50 g each), ensuring the dorsal musculature was preserved along the entire length of the spine and ribs. The left side of each fish was allocated for microbiological analysis, while the right side was designated for physicochemical and sensory analysis. This standardization was maintained throughout the 30-day experimental period. The fillets were stored in sterilized plastic bags and transported to the laboratory where they were stored in a B.O.D. (Biological Oxygen Demand) incubator at a controlled temperature of 4 ± 1 °C.

### 2.5. Microbiological Analysis

#### 2.5.1. Preparation of Dilutions

Under aseptic conditions, fragments representing the entire surface and depth of each fillet sample were randomly taken until reaching a total of 10 g. After weighing, 90 mL of 0.1% peptone water (HiMedia^®^ Laboratories, Kennett Square, PA, USA) was added, and the mixture was homogenized for approximately 60 s using a Stomacher-type device, resulting in the initial 10^−1^ dilution. Subsequently, serial dilutions of 10^−2^, 10^−3^, and 10^−4^ were made by taking a 1 mL aliquot from the initial dilution and transferring it to tubes containing 9 mL of 0.1% peptone water, continuing this process until reaching the 10^−4^ dilution.

#### 2.5.2. Most Probable Number of Total and Thermotolerant Coliforms

The Most Probable Number (MPN) method was used to estimate the quantity of total coliforms and thermotolerant coliforms in the samples according to APHA 9:2015 [12]. In the presumptive test, a 1 mL aliquot from the 10^−1^, 10^−2^, and 10^−3^ dilutions was inoculated into a series of three sterile tubes, each containing Lauryl Sulfate Tryptose (HiMedia^®^ Laboratories, USA) broth with inverted Durham tubes. The tubes were incubated for 48 h at 36 ± 1 °C. Tubes were considered positive if they showed gas formation in at least 1/10 of the Durham tube or effervescence when gently shaken and were selected for the confirmatory test. Confirmation of total and thermotolerant coliforms was performed by transferring a platinum loop of the positive tubes from LST to Brilliant Green Bile Lactose broth (HiMedia^®^ Laboratories, USA), incubated at 36 ± 1 °C for 48 h, and to EC broth (HiMedia^®^ Laboratories, USA), incubated at 45 ± 1 °C for 48 h in a water bath, respectively [12].

#### 2.5.3. Counts of Aerobic Mesophilic and Psychrotrophic Microorganisms

For the counting of mesophilic microorganisms, 1 mL from each dilution was inoculated into sterile Petri dishes, and 15–20 mL of Plate Count Agar (Kasvi^®^, Castelfidardo, Italy), previously melted and maintained at 46–48 °C, was added. The plates were mixed and incubated at 35 ± 1 °C for 48 h, according to the APHA 08:2015 method. After incubation, the plates were read, the number of colonies was multiplied by the corresponding dilution factor, and the result was expressed as colony-forming units per gram (CFU/g) [13]. The same procedure was used for counting psychrotrophic microorganisms, with the plates incubated at 7 ± 1 °C for 10 days, according to the APHA 13.61:2015 method [13].

#### 2.5.4. Counts of Coagulase-Positive Staphylococci

The method ISO 6888-1:1999/Amd 1:2003 was used for the enumeration of coagulase-positive staphylococci. Thus, 0.1 mL of each dilution was inoculated onto the surface of Baird-Parker agar (Kasvi^®^, Italy) supplemented with egg yolk solution and potassium tellurite (Dinâmica^®^, Itacoatiara, Brazil), using a Drigalski loop, and evenly spread until complete absorption. The plates were inverted and incubated at 35 ± 1 °C for 30 to 48 h. After bacterial growth, five typical colonies (shiny black with an opaque ring, surrounded by a clear, transparent halo distinct from the medium’s opacity) and atypical colonies (grayish or shiny black without a halo) were selected and transferred to Brain Heart Infusion (BHI) broth (Kasvi^®^, Italy) for biochemical tests (catalase, Gram, and coagulase). Subsequently, the Coagulase Test was performed using BHI culture tubes and lyophilized rabbit plasma (NewProv^®^, Pinhais, Brazil). These tubes were incubated in a water bath at 36 ± 1 °C for 24 h [14]. Readings were taken at 1, 2, 3, 4, and 24 h.

#### 2.5.5. Enterobacteriaceae Count

According to Mossel [15], MacConkey broth is used as a selective medium for the presumptive detection of bacteria from the Enterobacteriaceae family. Therefore, 1 mL from each dilution was inoculated into sterilized Petri dishes, and approximately 20 mL of MacConkey agar (Kasvi^®^, Italy), previously melted and maintained at 46–48 °C, was added. The inoculum was then homogenized with the medium. After the medium had completely solidified, the plates were incubated in an inverted position at 36 ± 1 °C for 18 to 24 h. Three to five typical colonies were selected and transferred to tubes containing BHI for Gram staining (as described above) and for the oxidase test [15].

### 2.6. Physicochemical Analysis

#### 2.6.1. Determination of pH

The pH was determined in duplicate in the epaxial muscle using a digital pH meter (Testo^®^ model 205, West Chester, PA, USA) equipped with a penetration electrode for direct insertion.

#### 2.6.2. Colorimetry

Color was determined using the CR-400 Chrome Meter (Konica Minolta Sensing Americas^®^, Inc., NJ, USA), which utilizes the CIELAB system (L*, a*, and b*) proposed by the Commission Internationale de l’Eclairage [16]. Parameters such as lightness (L*), redness intensity (a*), and yellowness intensity (b*) of the epaxial muscles were evaluated. The assessment was conducted at three different points on each muscle part to obtain an average value. Color intensity is expressed by chroma (*Cab*), while hue (*H°ab*) corresponds to the color name found in its pure state on the spectrum. These values were calculated using the formulas: *Cab* = √(*a*^2^ *+ b*^2^) and *H°ab* = arctan(b*/a*), according to Hernández et al. [17].

#### 2.6.3. Lipid Oxidation (TBARS)

For each sample, 5 g of fillet was weighed and placed in a Falcon tube for lipid oxidation analysis following the methodology described by Pikul et al. [18]. To the sample, 25 mL of 7.5% trichloroacetic acid (Synth^®^, Sao Paulo, Brazil) was added (one tube at a time, as the acid dries out the sample). The mixture was blended using a Turrax for 1 min and then filtered through another Falcon tube. All samples were processed in triplicate, and 5 mL of 0.01 M (thiobarbituric acid, Synth^®^, Brazil) was added to 5 mL of the extract. The mixture was then placed in a water bath at 100 °C for 40 min, with the test tube covered with marbles. The determination of TBARS (thiobarbituric acid reactive substances, expressed as mg of malondialdehyde/kg of sample) was performed using spectrophotometry at 532 nm.

#### 2.6.4. Sensory Analysis

Sensory quality assessments of the fillets were performed at each sampling interval by a panel of five trained evaluators, who were blinded to the treatment conditions. Panelists were trained over three 1-h sessions using fillets with known sensory deviations (e.g., loss of brightness, textural softening, off-odors), following adapted protocols based on Meilgaard et al. [19]. During training, panelists were calibrated using reference samples to ensure consistency in scoring brightness, firmness, appearance, and odor. Fish samples (50 g) from the dorsal region were presented individually to the panelists in individual booths under controlled conditions of light, temperature, and humidity. The panelists were asked to rate the characteristics of brightness, firmness, appearance, and odor on a scale from 1 to 10, with 10 being the best condition and 1 being the worst [8].

### 2.7. Statistical Analysis

Differences between treatments (10 g, 5 g, and control) and time (0, 7, 15, and 30 days) were determined using the non-parametric Kruskal-Wallis [20] test with GraphPad Prism version 9.0 software. Differences were considered significant when *p* < 0.05. Principal Component Analysis (PCA) was performed using PAST software (version 4.03) to explore patterns among microbiological (total and thermotolerant coliforms, mesophilic and psychrotrophic counts, *Staphylococcus* spp.), physicochemical (pH, TBARS, color parameters), and sensory variables over time and treatments. All variables were auto-scaled (mean-centered and standardized to unit variance) prior to analysis. Components with eigenvalues greater than 1.0 were retained based on the Kaiser criterion. The first two principal components (PC1 and PC2) were used to construct biplots and interpret sample distribution and variable correlations. Factor loadings were examined to identify the most influential variables in each component, allowing the identification of quality degradation patterns and treatment-specific effects during refrigerated storage. The PCA was used as a parameter to perform Spearman correlations (SAS 9.3)

## 3. Results

### 3.1. Microbiological Analysis

**Figure 1:**
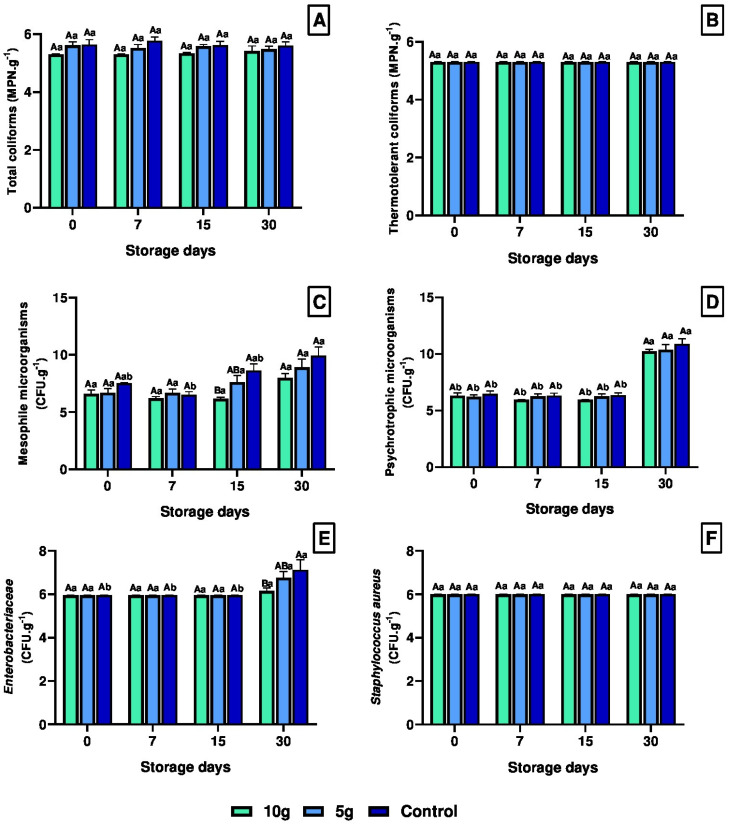
Mean values transformed into log(x + 5) (±SEM) for microbiological analysis. (**A**) Total coliforms; (**B**) Thermotolerant coliforms; (**C**) Mesophilic microorganisms; (**D**) Psychrotrophic microorganisms; (**E**) Enterobacteriaceae; (**F**) coagulase-positive staphylococci. Means (7 fish) followed by the same letter do not differ by the Kruskal-Wallis test (*p* < 0.05). Uppercase letters compare treatments within each experimental period, while lowercase letters evaluate the progression of each treatment across experimental periods. Sampling periods: 0, 7, 15, and 30 days post-slaughter (dps); Treatments: 10 g—treated with 10 g of *Chlorella*; 5 g—treated with 5 g of *Chlorella*; Control—untreated with *Chlorella*.

The results observed in the microbiological analysis of the fish samples are presented in Figure 1. The MPN counts for total coliforms showed significant decrease (*p* < 0.05) in fillets from tilapia treated with 10 g of *C. pyrenoidosa* after 15 days of storage (Figure 1A). The thermotolerant coliforms counts did not show significant differences (*p* ≥ 0.05) among treatments during 30 days of storage (Figure 1B).

Tilapia treated with *C. pyrenoidosa* exhibited a reduction in mesophilic microorganism counts throughout the shelf-life period (Figure 1C). Fillets from fish treated with 10 g showed a significant decrease in counts 15 days post-slaughter (dps) compared to the control group (Figure 1C). In control fish fillets, a significant increase in mesophilic microorganism counts was observed at 30 dps compared to the initial storage phase at 7 dps (Figure 1C). Psychrotrophic microorganism counts in tilapia fillets did not differ significantly (*p* ≥ 0.05) among treatments (Figure 1D). Microbiological analysis over time revealed a significant increase (*p* < 0.05) in psychrotrophic microorganism counts 30 dps in all treatments (Figure 1D).

The results for enterobacteria colony count showed a significant increase in the fillets from control tilapia 30 dps compared to fish treated with 10 g of *C. pyrenoidosa*, while the counts remained stable in the algae-treated groups (Figure 1E). The genus *Staphylococcus* was not detected in the tilapia fillets stored for 30 days at 4 °C (Figure 1F).

### 3.2. Physicochemical Analysis

**Figure 2:**
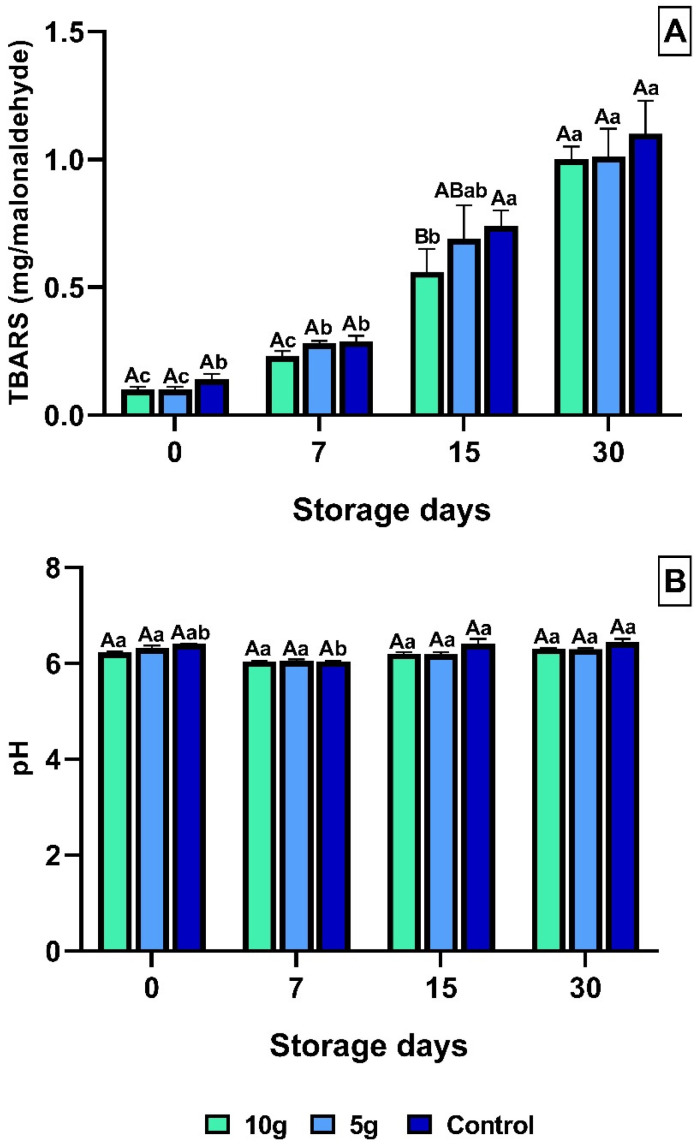
Analysis of TBARS and pH. (**A**) TBARS; (**B**) pH; Means (9 fish) followed by the same letter do not differ by the Kruskal-Wallis test (*p* < 0.05). Uppercase letters compare treatments within each experimental period, while lowercase letters evaluate the progression of each treatment across experimental periods. Sampling periods: 0, 7, 15, and 30 days post-slaughter (dps); Treatments: 10 g—treated with 10 g of *Chlorella*; 5 g—treated with 5 g of *Chlorella*; Control—untreated with *Chlorella*.

TBARS analysis (thiobarbituric acid reactive substances) over time revealed a significant increase (*p* < 0.05) in all treatments (Figure 2A). Fillets from tilapia treated with 10 g of *C. pyrenoidosa* showed lower values of TBARS throughout the shelf-life period, and these results were significantly lower when compared to those observed in the control group fillets at 15 dps (Figure 2A). During the fillet storage period, the pH presented the lowest values 7 dps for all treatments (Figure 2B). However, the fillets from the control group showed increased pH values at 15 and 30 dps (Figure 2B).

Correlation analysis between microorganism counts and pH or TBARS values revealed that control animals showed a significant (*p* = 0.0198 and *p* = 0.0197) positive correlation of 55.83% and 47.26% between the increase in the counts of mesophilic microorganisms and raising pH and TBARS values, respectively (Table 1).

#### Colorimetry

**Figure 3:**
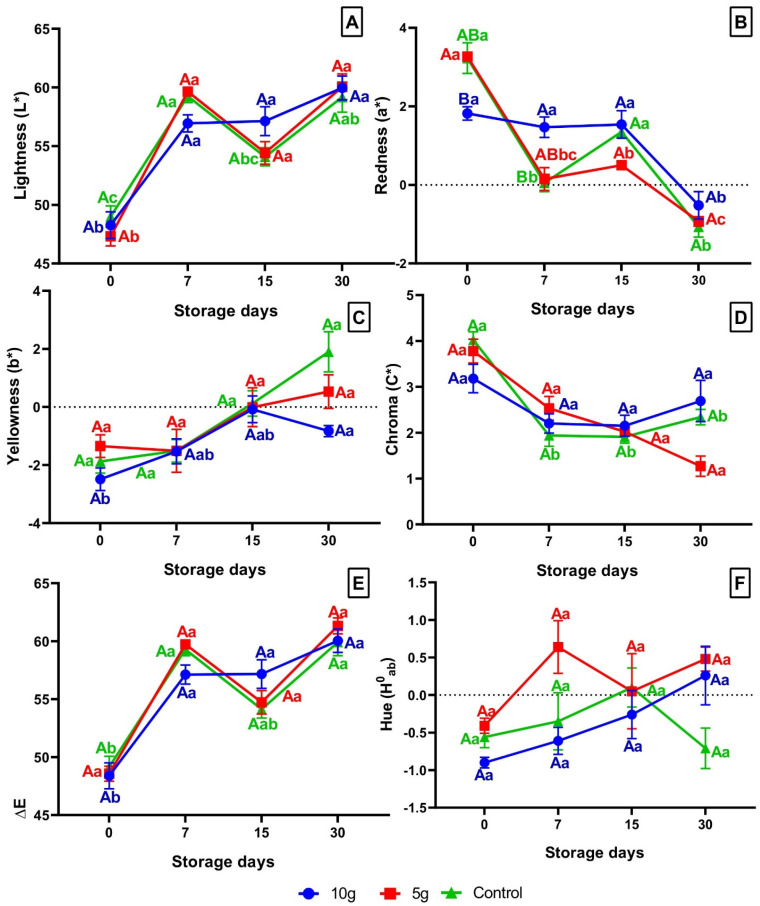
(**A**) Lightness (L*); (**B**) Redness (a*); (**C**) Yellowness (b*); (**D**) Chroma (C*); (**E**) Delta E (∆E); (**F**) Hue (H^0^_ab_). Means (9 fish) followed by the same letter do not differ by the Kruskal-Wallis test (*p* < 0.05). Uppercase letters compare treatments within each experimental period, while lowercase letters evaluate the progression of each treatment across experimental periods. Sampling periods: 0, 7, 15, and 30 days post-slaughter (dps); Treatments: 10 g—treated with 10 g of *Chlorella*; 5 g—treated with 5 g of *Chlorella*; Control—untreated with *Chlorella*.

In the colorimetry study of tilapia fillets, no significant changes (*p* ≥ 0.05) were observed in the analysis of luminosity (L*) and Delta E (∆E) in the comparison among different treatments (Figure 3A,E). It is worth highlighting in this analysis that there was a significant increase (*p* < 0.05) in both parameters during the refrigerated storage period in all treatments (Figure 3A,E). The analysis of red to green intensity (a*) showed that the fillets initially showed a tendency towards reddish coloration and turned green in all treatments, with these effects being less pronounced in fillets of fish treated with 10 g of green alga, which were statistically significant (*p* < 0.05) at 7 dps when compared to the other treatments (Control and treated with 5 g) (Figure 3B). Fillets from fish treated with 10 g exhibited an initial decrease (*p* < 0.05) in red color intensity (Figure 3B).

The analysis of the blue to yellow color intensity (b*) initially observed that the fillets of all treatments showed a tendency towards a bluish coloration that became yellowish with refrigerated storage, except for the fish fed with 10 g of *C. pyrenoidosa*, which presented a significantly different bluish color at 30 dps (Figure 3C). Chroma index was significantly reduced (*p* < 0.05) in tilapia fillets throughout the shelf-life period, except for fish fed 10 g of *C. pyrenoidosa* (Figure 3D). Fish treated with 5 g demonstrated a significant reduction (*p* < 0.05) in this index after 30 days compared to both the control group and fish fed 10 g (Figure 3D).

The analysis of the Hue angle (H^0^_ab_) over time did not show significant changes between fillets from different treatments throughout the storage period. However, control fish showed a significant decrease in this parameter at 30 dps when compared to fish treated with *C. pyrenoidosa* (Figure 3F). It is worth noting that fish treated with 5 g showed a significant increase in Hue at 7 dps when compared to control fish and fish treated with 10 g (Figure 3F).

Correlation analysis between microorganism counts and colorimetry (Table 2) revealed that fillets from control animals showed a significant (*p* = 0.0106 and *p* = 0.0305) positive correlation with the increase counts of psychrotrophic microorganisms and increase in b* (60.20%), as well as Delta E (∆E) (55.84%), respectively. Similar results were observed in fish treated with 5 g, which showed 54.68% positive correlation (*p* = 0.0430) between these same parameters (Table 2).

Correlation analysis between TBARS values and colorimetry revealed that fillets from control animals showed a significant (*p* = 0.0127) positive correlation with Delta E (∆E) (50.06%) and a significant (*p* = 0.0044) negative correlation with redness (a*) (−54.95%) (Table 3). Fillets from tilapia treated with 10 g of *C. pyrenoidosa* showed a positive correlation (*p* = 0.0255) of 45.50% between the increase in TBARS values to Hue pitch angle during storage (Table 3).

### 3.3. Sensory Analysis

**Figure 4:**
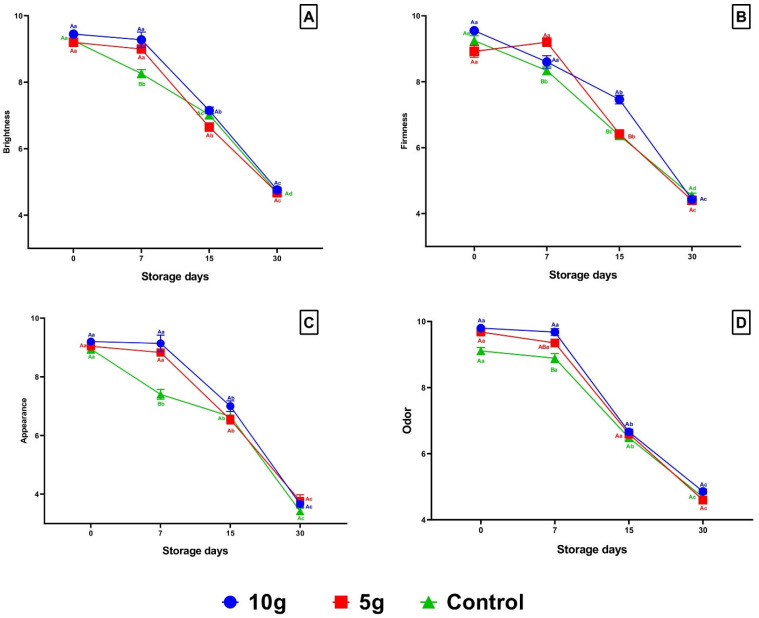
Sensory analysis. (**A**) Brightness; (**B**) Firmness; (**C**) Appearance; (**D**) Odor. Means (9 fish) followed by the same letter do not differ by the Kruskal-Wallis test (*p* < 0.05). Uppercase letters compare treatments within each experimental period, while lowercase letters evaluate the progression of each treatment across experimental periods. Sampling periods: 0, 7, 15, and 30 days post-slaughter (dps); Treatments: 10 g—treated with 10 g of *Chlorella*; 5 g—treated with 5 g of *Chlorella*; Control—untreated with *Chlorella*.

The results observed in the sensory analysis of the fish highlighted the following characteristics: brightness, firmness, appearance, and odor, as expressed in Figure 4. Over time, a significant worsening (*p* < 0.05) was observed in sensory parameters analyzed for all treatments, with these findings being more pronounced in control fish (Figure 4A–D). At 7 dps, a significant decrease in brightness, firmness, appearance and odor was observed in fillets of control fish when compared to fillets of fish treated with *C. pyrenoidosa* (Figure 4A–D).

### 3.4. Principal Component Analysis (PCA)

**Figure 5:**
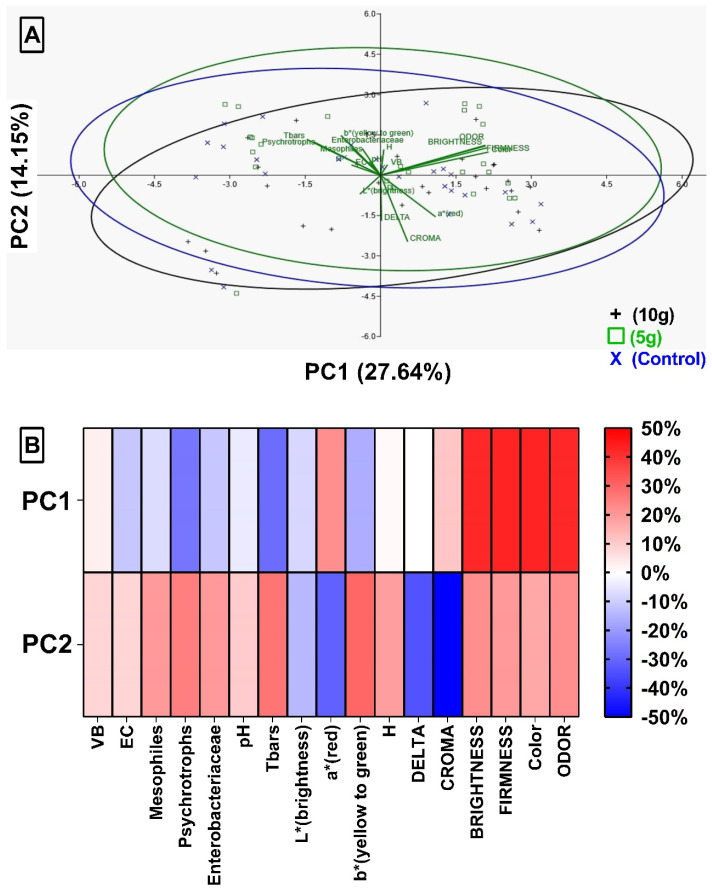
Principal component analysis (PCA) on the distribution of variables obtained after treatment of Nile tilapia with 10 g or 5 g of green alga (*Chlorella pyrenoidosa*) and control (untreated). (**A**) Relationship between variables studied by means of principal components. The directions of the vectors determine high scores in the respective variables. (**B**) Heat Map with the scores obtained in the three-dimensional PCA PC1 and PC2 of the variables used in the Spearman correlation test.

Principal component analysis (PCA) helps to understand the interactions (correlation) between the variables studied. Figure 5A shows the dispersion of the groups studied and the loads that each vector (variables) presents within the analysis. These loads are illustrated in the heat map (Figure 5B); the more intense the red color, the greater the load attributed to this variable within the analysis and the greater the correlation between these factors. The sum of components (PC) 1 and 2 explains 41.79% of the relationships between the variables studied.

These data show that there may be a correlation between the variables PC1 and PC2, as well as between themselves in the same component. Table 1 shows a positive correlation between pH and mesophiles (p2 = 55.83%) represented by low-intensity red colors. Similarly, the correlation between pH and psychrotrophics (p2 = 72.19%) can be observed, in which the intensity of red shown in Figure 5B is greater in psychrotrophics. It is interesting to note that the Delta E variable in Table 2 and Table 3 presents positive correlations with psychotrophics (p2 = 55.84%; 54.68%) and TBARS (p2 = 50.06%) respectively, even though their loadings tend to 0% (PC1) and −34% (PC2).

**Figure 6:**
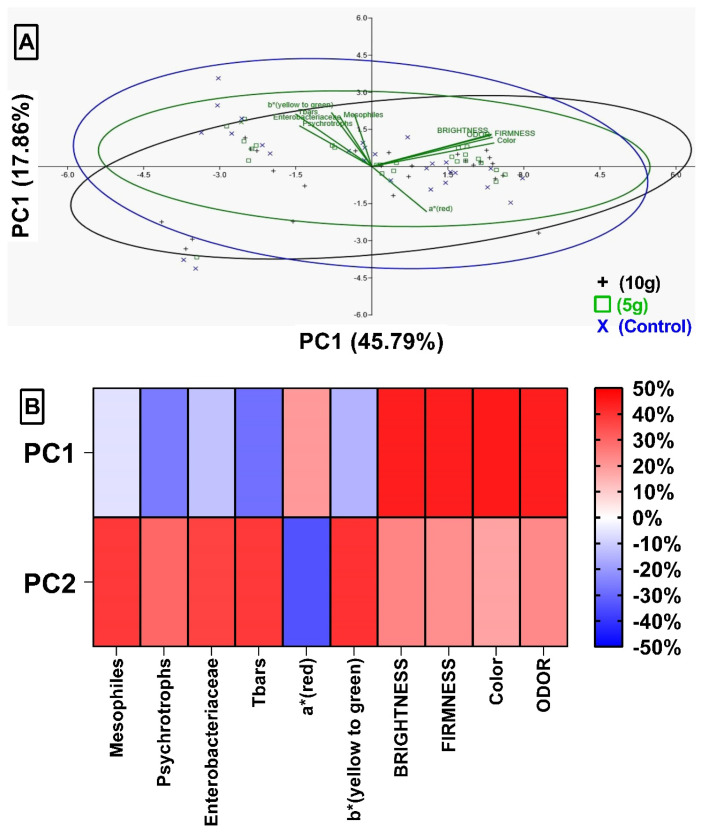
Principal component analysis (PCA) on the distribution of variables selected from the first PCA with loadings of 20% or more in PC1 and PC2 in Nile tilapia with 10 g or 5 g of green alga (*Chlorella pyrenoidosa*) and control (untreated). (**A**) Relationship between variables selected by principal components. The directions of the vectors determine high scores in the respective variables. (**B**) Heat Map with the scores obtained in the three-dimensional PCA PC1 and PC2 of the variables used in the Spearman correlation test.

In accordance with the results obtained in the loadings (the name given in the program) of the 17 variables studied in the first PCA (Figure 5), those that presented 20% or more (in PC1 or PC2) were selected. From this, a new PCA analysis was performed (Figure 6). This second analysis explains 63.65% (45.79% in PC1 and 17.86% in PC2) of the relationships between the data collected in the study. The heat map (Figure 6B) shows intense red colors in brightness, firmness, color and odor in the first principal component, PC1. In PC2, these tones appear in the bacteria evaluated during the study. The correlation between TBARS and mesophiles (p2 = 47.26%) presented in Table 1 can be observed with less intense staining (Figure 5B) and with greater intensity mainly in TBARS in Figure 6B.

**Figure 7:**
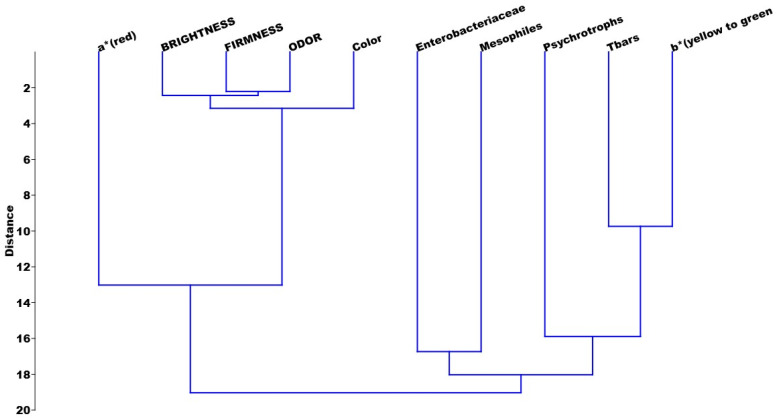
Clusters of loading variables with 20% or more in PC1 and PC2 in Nile tilapia with 10 g or 5 g of green alga (*Chlorella pyrenoidosa*) and control (untreated).

It is interesting to note that positive correlations with a high probability of significance can be observed when the variables present a higher load in the same component, such as psychotrophic vs. b* (Table 2). Similarly, observing the clusters (Figure 7), we can identify these correlations by the proximity (distance) in which the variables appear. Therefore, the greater the distance between them, the lower the probability of significance or even negative correlation coefficients (*p*^2^).

Table 3 shows a negative correlation between TBARS vs. a*. When observing the heat maps (Figure 5B and Figure 6B), the variable a* is colored pink (PC1) and blue (PC2). Therefore, when compared under the same main component or between the components, there is a large difference between them. This difference is evidenced when observing the distance between the variables in Figure 7.

## 4. Discussion

Nile tilapia supplemented with *C. pyrenoidosa* exhibited reduced microbial counts in fillets during a 30-day monitoring period, with an evident dose-response effect. Total coliforms and mesophilic bacteria count significantly increased in control fish after 15 days of refrigeration, in contrast to the lower counts observed in *C. pyrenoidosa*-treated fish. Total coliforms and thermotolerant coliforms are indicators used to assess the hygienic and sanitary conditions of food, and they can predict the presence of pathogenic microorganisms [3]. Junior et al. [21] emphasized the relevance of monitoring total and thermotolerant coliforms in Nile tilapia, noting a significantly higher prevalence and concentration of these indicators in fillet samples compared to whole fish. According to Ferreira et al. [22], the filleting process in tilapia production is predominantly manual, with limited mechanization. This extensive human handling increases the risk of cross-contamination, particularly by thermotolerant coliforms, due to direct contact with operators and surfaces. Although recent studies have demonstrated the ability of microalgae, including *C. pyrenoidosa*, to remove pathogens from wastewater [23], little is known about their antimicrobial activity. However, to date, no studies have been identified that assess the effects of supplementation with this alga in animal models and its influence on coliforms counts during the shelf life of other foods of animal’s origin.

Mesophilic and psychtrophic microorganisms are among the most widely used microbiological indicators of food quality, as they reflect the effectiveness of temperature and hygiene control during processing, transportation, and storage [24]. Stejskal et al. [25] reported that refrigerated hake fillets packaged with active films incorporating *Spirulina platensis* protein concentrate exhibited reduced counts of mesophilic, psychrotrophic, and Enterobacteriaceae microorganisms compared to control samples. These findings align with the results of the present study, in which tilapia supplemented with the green alga *C. pyrenoidosa* demonstrated similarly decreased levels of these microbial groups during refrigerated storage. In a study on the shelf life of tilapia fillets treated with astaxanthin, a carotenoid produced naturally in another freshwater microalga, *Haematococcus pluvialis*, Aracati et al. [8] reported significant reductions in mesophilic and psychrotrophic bacterial counts, attributed to the compound’s antioxidant activity. Similar outcomes were reported by Santos et al. [26], who evaluated smoked tilapia fillets coated with chitosan. Furthermore, *C. pyrenoidosa* has demonstrated in vitro antimicrobial and antifungal activity [27], supporting the results of the present study, which showed a reduction in mesophilic and Enterobacteriaceae counts in fillet samples supplemented with *C. pyrenoidosa* at 5 g and 10 g doses, with the most pronounced effect observed at 10 g.

No *Staphylococcus* spp. counts were detected in the tilapia fillets stored for 30 days at 4 °C in the present study. The absence of these microorganisms suggests that the fillets were clean and safe, and that slaughter was performed under good hygienic and sanitary practices, corroborating the study by Atitallah et al. [28]. Coagulase-positive *Staphylococcus*, such as *Staphylococcus aureus*, are responsible for producing a heat-stable toxin and are one of the most prevalent foodborne pathogens [21]. Humans are common carriers of this microorganism, and the authors isolated multiple strains of *Staphylococcus aureus* from the nasal mucosa of food handlers [29], reinforcing the findings of Junior et al. [21], who demonstrated that the presence of *S. aureus* in Nile tilapia may be attributed to improper handling, inadequate hygiene practices, storage deficiencies, and cross-contamination.

Lipid oxidation is one of the main factors contributing to the deterioration of food quality, especially in fish flesh, directly influencing color changes, undesirable flavor, nutritional value, and shelf life [30]. Tilapia fillets from fish supplemented with *C. pyrenoidosa* exhibited significantly lower TBARS levels in comparison to fillets from the control group. Algae contain antioxidant compounds that are efficient oxygen radical scavengers and can diminish the oxidation of fatty acids [4]. The application of an algal-based coating containing *Spirulina platensis* and *Chlorella vulgaris* to refrigerated veal fillets was investigated by Shafiei and Mostaghim [31], who observed a marked decrease in TBARS values, reflecting enhanced oxidative stability in the treated samples. The findings of Takyar et al. [4], who emphasized the antioxidant potential of these algae in delaying lipid oxidation, are consistent with those of the present study, which likewise demonstrated a pronounced reduction in lipid oxidation throughout the storage period.

A positive correlation between the increase in mesophilic bacterial counts and both TBARS levels and pH values was observed exclusively in fillets from the control tilapia group. Elevated pH during storage is known to compromise food quality by facilitating microbial growth, as bacteria metabolize low-molecular-weight compounds present in fish muscle tissue, contributing to pH elevation [8]. The decrease in pH observed during the initial days of storage may be attributed not only to post-mortem glycolysis and residual glycogen degradation but also to the accumulation of low molecular-weight compounds produced by microbial and enzymatic activity. These include organic acids (e.g., lactic and acetic acids), free amino acids, and other small metabolites, which contribute to acidification of the muscle tissue. Over time, as proteolytic degradation progresses, the formation of basic compounds such as ammonia and trimethylamine may lead to pH stabilization or a slight increase, depending on the balance of microbial populations and metabolic pathways [32,33]. These alterations were not detected in fillets from fish supplemented with *C. pyrenoidosa*, suggesting a potential antioxidant effect of the dietary supplementation in enhancing fillet stability under refrigerated conditions. Additionally, our findings, which showed a decline in pH levels across all experimental groups by day 7 of refrigerated storage compared to day 0 (slaughter day), are consistent with previous reports indicating that live fish typically exhibit a natural pH slightly above 7.0, which decreases significantly post-mortem due to rigor mortis and the accumulation of lactic acid resulting from glycogen metabolism [34].

Color plays a crucial role in the acceptance of food products, acting as a key indicator of quality, freshness, preservation status, flavor perception, and commercial appeal. In the present study, both luminosity (L) and total color difference (∆E) values increased over the storage period across all treatment groups. This trend may be associated with the progressive darkening of the fillets during refrigeration, as the initially fresh fish with high luminosity underwent deterioration, resulting in a gradual loss of brightness [8]. In fish muscle, particularly in Nile tilapia (*Oreochromis niloticus*), the colorimetric parameters lightness (L*), redness (a*), and yellowness (b*) are influenced by the deposition of both endogenous and dietary pigments. The redness (a)* value is strongly associated with the presence of carotenoids such as astaxanthin, β-carotene, and canthaxanthin, which can be absorbed from the diet and deposited in the muscle tissue, enhancing reddish hues. These pigments are lipophilic and accumulate primarily in the subcutaneous and intramuscular fat, influencing a* values through their antioxidant and color-enhancing effects [7,35]. The yellowness (b)* component is also affected by carotenoids, particularly lutein and zeaxanthin, which impart yellow-orange tones to the flesh. Meanwhile, lightness (L)* is less directly affected by specific pigments and more influenced by muscle structure, water and fat content, and the degree of lipid oxidation, all of which alter the light scattering properties of the muscle. However, high concentrations of deposited pigments may contribute secondarily to light absorption and reflectance. In this study, supplementation with *Chlorella pyrenoidosa*, a microalga rich in chlorophylls and carotenoids, likely contributed to the observed variations in a* and b* values in the fillets, especially in the 10 g/kg group, indicating a dose-dependent enhancement of pigment-related color stability.

In the present study, fillets from tilapia treated with 5 g of *C. pyrenoidosa* and those from the control group exhibited a more intense green coloration (−a*). In contrast, fillets from fish supplemented with 10 g of *C. pyrenoidosa* showed a less pronounced green hue (−a) only after 30 days of storage. The predominance of green coloration (−a*) observed at the end of the storage period was associated with loss of fillet quality. A negative correlation between increased TBARS levels and decreased redness intensity (a* values) was observed in both the control group and the 5 g supplementation group. This effect was less evident in fillets from tilapia fed 10 g of *C. pyrenoidosa,* suggesting a dose-dependent protective role of the alga against lipid oxidation-related color loss. These findings are consistent with those reported by Aracati et al. [8] in tilapia fed astaxanthin, and by Majdinasab et al. [36] in rainbow trout supplemented with alginate.

An increase in the yellow component (+*b*) was observed throughout the shelf life in the group supplemented with 10 g of *C. pyrenoidosa*. According to Alfaia et al. [37], the inclusion of *C. vulgaris* in broiler diets led to a yellowish coloration in breast and thigh muscles, attributed to the high carotenoid content of this alga. However, An et al. [38] reported that low inclusion levels (0.05%, 0.15%, and 0.5%) of *Chlorella* in broiler diets did not significantly affect breast meat coloration. These findings help explain the yellowing observed in fillets from fish supplemented with 10 g of *C. pyrenoidosa*, whereas this effect was not evident in the 5 g supplementation group over the 30-day storage period. An increase in the yellow component (+b) and total color difference (∆E) was positively correlated with the rise in psychrotrophic microbial counts in control fish fillets. These results suggest that the observed colorimetric changes also were associated with the proliferation of psychrotrophic microorganisms capable of growing under refrigeration, thereby compromising fillet quality.

Chroma (C) values decreased during refrigerated storage, indicating a loss in color intensity. This finding is consistent with previous studies by Aracati et al. [8] and Hernández et al. [17], who investigated the shelf life of *Oreochromis niloticus* and *Argyrosomus regius* fillets, respectively, and also reported this alteration during the shelf life. In contrast, the hue angle tended to shift toward a more reddish tone, which may be attributed to the presence of carotenoids, as *C. pyrenoidosa* is known to be a rich source of these pigments. Fradique et al. [39] reported that *C. vulgaris* accumulates substantial amounts of carotenoids in muscle tissue. Regarding total color difference (∆E), both groups showed an increase over time, with more pronounced changes observed in the control group. Additionally, tilapia fed with *C. pyrenoidosa* exhibited increased hue angle values (*H°ab*), indicating a shift toward a more reddish coloration. This observation is consistent with previous findings in *O. niloticus* and *Pagrus pagrus* fed with astaxanthin [8,40]. However, in fish supplemented with 10 g of *C. pyrenoidosa*, the increases in *H°ab* values were positively correlated with elevated TBARS levels, suggesting a relationship between lipid oxidation and color alteration. Mattje et al. [40] also reported that storage duration affects hue, with food products tending to become more yellowish by the end of refrigerated storage, primarily due to oxidative processes. Fish flesh is more perishable compared to red meat and poultry due to high levels of free amino acids, volatile basic nitrogen, and polyunsaturated fatty acids (PUFAs) [4]. Oxidation of PUFAs leads to the deterioration of food quality and consequently reduces shelf life, causing unpleasant and rancid flavors due to the formation of aldehydes and ketones, and diminishing nutritional value through the destruction of essential fatty acids and fat-soluble vitamins. Additionally, it can have adverse health effects due to the formation of free radicals [3]. The addition of algae extracts to fish has antioxidant activity capable of extending shelf life while preserving quality. Thus, *C. pyrenoidosa* can delay lipid oxidation and maintain the sensory properties of refrigerated fish [4]. Sensory analysis results (brightness, firmness, color, and odor) showed that overall acceptability ratings were significantly higher in algae-treated tilapias compared to control animals. Furthermore, a gradual reduction in sensory parameters was observed over time in all groups, with more pronounced effects in the control group, indicating a reflection of fillet quality loss. Lópes-Cánova et al. [41] described similar sensory changes in tilapia during refrigerated storage, including a gradual increase in pH values, corroborating the findings of this study.

The results from microbiological, physicochemical, and sensory evaluations highlight the beneficial role of *C. pyrenoidosa* in extending the quality of refrigerated tilapia fillets. Both the 5 g and 10 g doses improved microbial stability, sensory attributes, and reduced lipid oxidation over 30 days, with the higher dose producing more pronounced effects, suggesting a dose–response correlation. While this study demonstrated the beneficial effects of *Chlorella pyrenoidosa* supplementation on the quality of tilapia fillets, several limitations should be addressed in future research. Detailed chemical profiling of the microalga (e.g., chlorophylls, carotenoids, antioxidants) is needed to clarify the active compounds responsible for the observed effects. Inclusion of a reference antioxidant (e.g., vitamin E) and molecular microbiological tools (e.g., 16S rRNA sequencing) would strengthen causal inference. Moreover, assessing the economic feasibility, nutritional composition of fillets, and storage conditions that reflect commercial realities (e.g., MAP, temperature variation) would enhance practical relevance. Although *C. pyrenoidosa* is generally recognized as safe (GRAS), monitoring for potential contaminants remains essential to ensure food safety.

## 5. Conclusions

The dietary use of *Chlorella pyrenoidosa* proved effective in enhancing the microbiological stability, oxidative resistance, and sensory quality of Nile tilapia fillets stored under refrigeration. Incorporating this alga into the fish diet offers a practical strategy to naturally extend fillet shelf life and improve product quality, with potential applications in sustainable aquaculture and value-added fish processing.

## Figures and Tables

**Table 1:** Correlation analysis between microorganism counts and pH or TBARS values observed in the tilapia fillets treated or not with *C. pyrenoidosa*.

Correlated	Experimental Sampling ^1^	Correlation Analysis	
Parameters		ρ ^2^	Prob. > |ρ| ^2^
pH X Mesophilic microorganisms	Control	0.5583	0.0198
	5	−0.1182	0.6626
	10	0.4849	0.0793
TBARS X Mesophilic microorganisms	Control	0.4726	0.0197
	5	0.3094	0.1508
	10	0.1261	0.5662

^1^ Correlation between control animals (*n* = 28); 5—animals treated with 5 g *C. pyrenoidosa*/kg of feed (*n* = 28); 10—animals treated with 10 g *C. pyrenoidosa*/kg of feed (*n* = 28). ^2^ ρ = Coefficient of Spearman Correlation; Prob. > |ρ|—Significance Probability of ρ value.

**Table 2:** Correlation analysis between microorganism counts and colorimetry observed in the tilapia fillets treated or not with *C. pyrenoidosa*.

Correlated	Experimental Sampling ^1^	Correlation Analysis	
Parameters		ρ ^2^	Prob. > |ρ| ^2^
Psychrotrophic microorganisms X b*	Control	0.6020	0.0106
	5	0.3410	0.1204
	10	0.3450	0.1255
Psychrotrophic microorganisms X ∆E	Control	0.5584	0.0305
	5	0.5468	0.0430
	10	0.1286	0.5785

^1^ Correlation between control animals (*n* = 28); 5—animals treated with 5 g *C. pyrenoidosa*/kg of feed (*n* = 28); 10—animals treated with 10 g *C. pyrenoidosa*/kg of feed (*n* = 28). ^2^ ρ = Coefficient of Spearman Correlation; Prob. > |ρ|—Significance Probability of ρ value.

**Table 3:** Correlation analysis between TBAR’s values and colorimetry in the fillet of tilapia treated or not with *Chlorella pyrenoidosa*.

Correlated	Experimental Sampling ^1^	Correlation Analysis	
Parameters		ρ ^2^	Prob. > |ρ| ^2^
TBARS X H^0^_ab_	Control	0.0523	0.8036
	5	0.3374	0.1577
	10	0.4550	0.0255
TBARS X ∆E	Control	0.5006	0.0127
	5	0.3405	0.1811
	10	0.3693	0.0757
TBARS X a*	Control	−0.5495	0.0044
	5	−0.5539	0.0092
	10	−0.2962	0.1598

^1^ Correlation between control animals (*n* = 28); 5—animals treated with 5 g *C. pyrenoidosa*/kg of feed (*n* = 28); 10—animals treated with 10 g *C. pyrenoidosa*/kg of feed (*n* = 28). ^2^ ρ = Coefficient of Spearman Correlation; Prob. > |ρ|—Significance Probability of ρ value.

## Data Availability

The original contributions presented in the study are included in the article. Further questions can be directed to the corresponding author.
